# The Evolution of Nitric Oxide Function: From Reactivity in the Prebiotic Earth to Examples of Biological Roles and Therapeutic Applications

**DOI:** 10.3390/antiox11071222

**Published:** 2022-06-22

**Authors:** Mark Shepherd, Daniela Giordano, Cinzia Verde, Robert K. Poole

**Affiliations:** 1 School of Biosciences, RAPID Group, University of Kent, Canterbury CT2 7NJ, UK; m.shepherd@kent.ac.uk; 2 Institute of Biosciences and BioResources (IBBR), CNR, Via Pietro Castellino 111, 80131 Napoli, Italy; daniela.giordano@ibbr.cnr.it (D.G.); cinzia.verde@ibbr.cnr.it (C.V.); 3 Department of Marine Biotechnology, Stazione Zoologica Anton Dohrn (SZN), Villa Comunale, 80121 Napoli, Italy; 4 School of Biosciences, Firth Court, The University of Sheffield, Sheffield S10 2TN, UK

**Keywords:** evolution, cell signalling, toxicity, origin of life

## Abstract

Nitric oxide was once considered to be of marginal interest to the biological sciences and medicine; however, there is now wide recognition, but not yet a comprehensive understanding, of its functions and effects. NO is a reactive, toxic free radical with numerous biological targets, especially metal ions. However, NO and its reaction products also play key roles as reductant and oxidant in biological redox processes, in signal transduction, immunity and infection, as well as other roles. Consequently, it can be sensed, metabolized and modified in biological systems. Here, we present a brief overview of the chemistry and biology of NO—in particular, its origins in geological time and in contemporary biology, its toxic consequences and its critical biological functions. Given that NO, with its intrinsic reactivity, appeared in the early Earth’s atmosphere before the evolution of complex lifeforms, we speculate that the potential for toxicity preceded biological function. To examine this hypothesis, we consider the nature of non-biological and biological targets of NO, the evolution of biological mechanisms for NO detoxification, and how living organisms generate this multifunctional gas.

## 1. Introduction: The Chemical Nature of Nitric Oxide and Its Biological Derivatives

### 1.1. NO as a Radical

The biological importance of NO is rooted in its unique chemical properties. The chemistry of NO and derived and related species (“congeners”) is complex, both in non-biological and biological environments, due to the multitude of species that can be generated from NO, particularly in complex biological milieu and the numerous possible reaction targets of these derived species (for an overview, see [1]). Importantly, the fate of NO is always a function of this (bio)chemical environment. The redox relationship between NO and the nitrogen oxides (NOx) that may be formed from it is discussed in [2]. Critically, NO has an unpaired electron and is, therefore, considered a free radical. However, the unpaired electron is not associated solely with the nitrogen atom of NO, but rather, is delocalized throughout the molecule in a π* antibonding orbital.

Although the term free radical is often associated with extreme reactivity (e.g., having strong oxidant activity), this is not the case with NO, as evidenced by its relatively low reduction potential (E° = −0.55 V for the NO, H^+^/HNO couple [2]). Thus, NO is a poor one-electron oxidant and is also poor at H-atom abstraction. However, NO rapidly reacts with existing radicals. Thus, NO is a potent antioxidant capable of quenching otherwise deleterious/oxidizing radical chemistry and reacts with dioxygen (O_2_), generating nitrogen dioxide (NO_2_). Like NO, NO_2_ is itself a free radical species but a fairly strong oxidant, as indicated by a reduction potential of +1.04 V for the NO_2_/NO_2_^−^ couple [3]. Therefore, the reaction of NO with O_2_ takes two relatively weak oxidants (the reduction potential for the O_2_/O_2_^−^ couple is −0.33 V) and forms a reasonable one-electron oxidant. These considerations are probably important in considering the evolution of NO-related chemistry and biochemistry in the early biosphere, since NO behaviour would have been affected by the presence of photosynthetically derived O_2_, which probably appeared in the Earth’s atmosphere after NO (see Section 2).

### 1.2. Fates of NO in Biology

NO_2_ produced from NO in the presence of O_2_ also oxidizes diverse biologically relevant functional groups, such as cysteine thiols, tyrosine residues and polyunsaturated fatty acids. In the absence of these NO_2_-reactive species (i.e., reductants), the fate of NO_2_ generated via the reaction of NO with O_2_ is to react with another NO (since they are both radicals) to give dinitrogen trioxide (N_2_O_3_). N_2_O_3_ (which is not a radical) is electrophilic and, in aqueous systems, will react with H_2_O to give two equivalents of nitrite (NO_2_^−^). If other nucleophiles are present (e.g., thiols, amines, etc.), they can be nitrosated by N_2_O_3_.

These complex recombinations can result in the NO-mediated nitrosation of biological nucleophiles, generating, for example, nitrosothiols (RSNO) of great biological significance [4]. Note that the terms *S*-nitrosation and *S*-nitrosylation are terms that are often used interchangeably. By definition, the process of nitrosation involves the transfer of the NO^+^ group from a nitrosating agent to a nucleophilic receptor, such as a thiol (for references, see [5]). NO cannot, therefore, itself react as a nitrosating agent, unless there are oxidizing agents present, such as a transition metal species or O_2_. Thus, NO cannot nitrosate thiols. Potential nitrosating agents of biological relevance are *S*-nitrosothiols (SNOs) or iron nitrosyls containing NO^+^ nitrosyl groups. The reactivity of the SNO and the nucleophilic substrate are important in determining the rate of reaction. Thiolates are nitrosated readily to give SNOs, which may in turn function as trans-nitrosating agents towards more reactive nucleophiles. Alternatively, they may act as releasers of NO under other conditions. However, *S*-nitrosylation is a term that may be used when there is ambiguity regarding the process that leads to SNO formation. Further to this, a “nitroso” species is one where an NO functional group is bound to another atom or molecule, whereas “nitrosyl” is used to describe a metal species bound by NO.

In mammalian systems, oxidation appears to be the major fate of NO. However, reduction of NO is well-established in prokaryotes. One-electron reduction of NO generates nitroxyl (NO^−^/HNO) [6,7]).

Reaction with metal centres is a major feature of NO chemistry. As described below (Section 4), NO reacts with O_2_-bound metal species such as oxyhaemoglobin or oxymyoglobin to form NO_3_^−^ in a reaction that is central to NO detoxification by the flavohaemoglobins. Along with its reaction with O_2_ and O_2_-derived species, NO also binds metals directly. Most notable in biological systems is the reaction of NO with haemproteins, although NO can bind other non-haem metalloproteins as well. A major site of action of NO in mammalian systems is the haemprotein soluble guanylate cyclase (sGC), which binds NO via its ferrous haem, leading to the formation of a five-coordinate ferrous nitrosyl species that is presumably responsible for enzyme activation. Significantly, when O_2_ and CO bind most ferrous haems, a six-coordinate geometry is preferred, making NO a unique ligand among small molecule diatomic signalling species. Again, the chemistry of NO [8] is fundamental to its biological properties and, presumably, the evolution in primordial lifeforms of NO-binding sites.

The above brief discussion of NOx and the associated chemical descriptions cannot be comprehensive but serves as a starting point for understanding the diversity and complexity of biological NO chemistry. For more complete descriptions of these and other aspects of NOx chemistry, readers are encouraged to find one of the available reviews (for example, [2,9,10]).

## 2. Emergence of NO in the Earth’s Atmosphere

It is widely accepted that NO was probably first generated by volcanic activity, lightning discharge reactions [11,12] and, to a lesser extent, coronal discharges. These events produced NO from CO_2_ and N_2_ in the Hadean and Archaean eras (that is, from 4.5 billion years ago (bya) to 2.5 bya; 1 billion = 10^9^) [13,14]. This was followed by the subsequent formation of atmospherically produced NO_3_^−^ and NO_2_^−^, which rained into the oceans and are the most attractive high-potential candidates for electron acceptors for subsequent lifeforms, that is, chemicals that “pull” and enable crucial redox reactions of autotrophic metabolism, notably in submarine alkaline hydrothermal vents [15]. Wong and colleagues [15] have quantified the levels of NOx produced through lightning and photochemical processes in the atmosphere of the Hadean eon (i.e., beginning with the formation of the Earth and preceding the Archean eon when the Earth’s crust had cooled sufficiently for continents to form and microbial lifeforms to evolve). Furthermore, photochemical reactions involving NO and water vapour are considered to have produced acids such as HNO, HNO_2_, HNO_3_ and HO_2/_NO_2_, which rained into the ocean, generating more nitrate (NO_3_^−^) and nitrite (NO_2_^−^). Wong et al. calculate that these NO_x_ fluxes are expected to produce sufficient (micromolar) ocean concentrations of high-potential electron acceptors for the emergence of life after only tens of thousands to tens of millions of years. Note that the reaction of NO with superoxide (O_2_^−^) generates peroxynitrite (ONOO^−^), which will eventually decompose to generate more nitrate.

**Figure 1:**
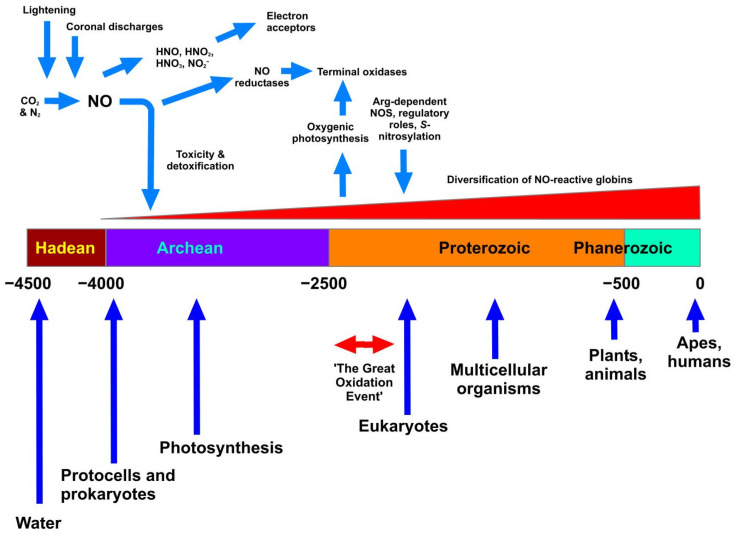
The appearance of NO on Earth and relationships with geological time. The Figure shows as a horizontal bar the division of time into the accepted eons (Hadean through to Phanerozoic). The units are in millions of years, and the scale ranges from 4.5 bya (4.5 × 10^9^ years) to the present. Blue vertical arrows show accepted, but approximate, timings of the appearance or prevalence of the most significant biological events. Haemoglobins are considered to have emerged alongside the appearance of single-celled life, with an initial role in NO detoxification that later evolved and diversified (see text). At the top, NO is shown as being formed under the influence of lightning and coronal discharges in the Hadean atmosphere, subsequently generating various NOx, which served as early electron acceptors. NO reductase probably appeared before the related terminal oxidases, which appeared after the Great Oxidation Event. All timings are approximate and solely illustrative.

Figure 1 illustrates these events in approximate, and estimated, relationship to geological time scales. We presume that the appearance of NO in the Archean era would create a potentially inhospitable scenario for the earliest lifeforms. This may therefore be coincident with the earliest appearance of haemoglobins, whose earliest, primary function is likely to have been, not O_2_ binding, but reaction with NO and consequent detoxification via conversion to nitrate. Vinogradov et al. [16] favours the view that a globin having a single domain structure was the ancestral globin. The next stage comprised a splitting to give single-domain “2-over-2” globins (referring to the intramolecular organisation of protein helices) and sensor-like globins, followed by the covalent addition of C-terminal reductase domains resulting in the chimeric flavohaemoglobin (e.g., Hmp) and globin-coupled sensors. Many globin classes, but especially the flavohaemoglobins of various prokaryotic and eukaryotic microbes, are effective in haem-mediated NO conversion to nitrate. The last stage encompassed the lateral gene transfers of some members of the three globin lineages to specific groups of Archaea and Eukaryotes.

Other gasotransmitters that we now consider central to signalling in biology, i.e., primarily carbon monoxide (CO) and hydrogen sulfide (H_2_S), were also surely present in the prebiotic atmosphere; thus, life evolved with these gases [17]. Nitrogen is cycled in our contemporary atmosphere by intimately interlinked biotic processes (see Section 3.1). However, before any organisms emerged, all nitrogen cycling must have been abiological; indeed, this cycling may have set the stage for the origin of life.

To understand how nitrogen cycling might proceed on terrestrial planets with comparable geodynamic activity to Earth, but on which life did not arise, Laneuville and coworkers [18] constructed a kinetic model of nitrogen cycling in its various major chemical forms (e.g., N_2_, reduced (NHx) and oxidized (NOx) species) between major planetary reservoirs (the atmosphere, oceans, crust and mantle) and included inputs from space. The model predicts a significant increase in oceanic nitrogen content over time, mostly as NHx, while atmospheric N_2_ content was lower than today. These distributions may have contributed to nitrogen assimilation and cycling over geological time scales.

With the Great Oxidation Event [19,20], which produced a rapid rise in the partial pressure of atmospheric O_2_ between 2.45 and 2.22 bya, cells began to utilize gases in a variety of physiological functions for signalling purposes [17]. The presence of O_2_ would have enabled reactions with NO, as described in Section 1. Atmospheric O_2_ concentrations reached modern levels in the Devonian period as a result of photosynthesis [21], and with the consequent change in the oxidation state of the atmosphere, H_2_S was eliminated as a primary electron donor, and NO and CO became less advantageous as electron acceptors [22].

At some point in evolution, NO became an ancestral regulator of diverse metabolic processes. In particular, *S*-nitrosylation of protein cysteine residues emerged as a preeminent effector of NO bioactivity [23], but the evolutionary pressures that drove this are unknown, as is the timing of the appearance of cellular NO synthases (NOSs).

Overall, we can speculate that complex chemical reactions between NO, O_2_, CO and H_2_S preceded the evolution of life, but discerning how the NO-binding sites we discuss below may have contributed to the evolution of enzymes and other biomolecules is extremely challenging.

## 3. Evolution of Biotic Routes for NO Production

Since its appearance in the Earth’s atmosphere, NO is likely to have played a crucial role in the early stages of the evolution of life. Feelisch and Martin [24] suggest that NO may have played a role as a critical defence mechanism for ancestral microorganisms at a time when life faced the problem of rising atmospheric levels of ozone (O_3_) formed from photolysis of O_2_. Biotic production of NO may have allowed extracellular neutralization of toxic O_3_, thus acting as a protective mechanism against oxidative damage, with evolutionary benefits from natural selection before the appearance of specific electron-accepting enzymes. Pathways of NO formation may then consequently have developed further to serve other useful functions. Although mammalian cells produce NO from L-arginine, the origin of this ability might have arisen from the essential process of either nitrification or denitrification in prokaryotic cells or, alternatively, from an endogenous L-arginine–NO pathway [24].

### 3.1. The Biological Nitrogen Cycle for NO

The major cycles relating to NO biochemistry are summarized in Box 1.

Box 1 **The major cycles of NO biochemistry.**

1. The citrulline–NO cycle

NO is generated from L-arginine that is produced from L-citrulline via the action of two of the five urea cycle enzymes. Briefly, argininosuccinate synthetase catalyses the rate-limiting step, converting L-citrulline to argininosuccinate, which is then converted to L-arginine by argininosuccinate lyase. The L-arginine so formed is then converted to NO and L-citrulline by the action of eNOS to complete the cycle [25].

2. Myoglobin- and haemoglobin-catalysed reactions of NO

Globins catalyse important reactions with nitrogen oxide species, such as NO dioxygenation and nitrite reduction. The formation of NO from nitrite is a reaction catalysed by globins that has received increasing attention due to its potential as a hypoxic NO signalling mechanism. NO dioxygenation is by far the most common reaction of NO with the haem group of globins in nature, and sometimes constitutes the main function of the protein, as in the case of flavohaemoglobins [26].

NO appeared on Earth long before the living organisms that now markedly contribute to the cycling of nitrogen species, including NO. In brief, dead organic matter in soils is mineralized to give ammonium ions, which can be used by soil microbes and plants. However, there is increasing evidence that chemical reactions alone can significantly affect the terrestrial nitrogen cycle, which was previously assumed to be mainly dominated by biological processes [27]. With that caveat, one component of the global nitrogen cycle that is of special significance here is the microbial release of NO into the atmosphere from soils. One such mechanism is denitrification, in which bacteria switch from O_2_-terminated respiration to nitrate respiration. Overall, NO_3_^−^ is converted via NO_2_^−^, NO and nitrous oxide (N_2_O) to dinitrogen (N_2_). Four essential reductases that sequentially reduce nitrate (*nar* genes), nitrite (*nir* genes), nitric oxide (*nor* genes) and nitrous oxide (*nos* genes) to dinitrogen are involved. Microbial denitrification has long been identified as a source of nitrogen trace gases under reducing conditions, whereas their production during nitrification is far from completely understood. Numerous reviews cover these processes in depth [28,29,30]. In brief, bacteria generate NO via many mechanisms, some of which are still unclear. Anaerobic denitrifying bacteria reduce NO_3_^−^ to dinitrogen gas with NO as an intermediate [30,31], or NO may be formed during a short-circuit in the nitrogen cycle in which commensal bacteria catalyse the anaerobic reduction of NO_3_^−^ via NO_2_^−^ to ammonia [32]. Minor sources of NO could arise from the chemical reduction of NO_2_^−^ by ferrous ions or the reduced forms of cytochromes and iron–sulfur centres in anaerobic environments [33]. However, the activity of NO reductase might be expected to render this route for NO release insignificant.

However, abiotic processes also significantly contribute to the formation of gaseous nitrogen-containing products in soil. Several reactions that involve the nitrification intermediates, NO_2_^−^ and hydroxylamine (NH_2_OH), are known to produce NO and N_2_O. The most significant abiotic reactions include self-decomposition of NO_2_^−^, the reactions of NO_2_^−^ with reduced metal cations, nitrosation of soil organic matter, the reaction between NO_2_^−^ and hydroxylamine, and the oxidation of hydroxylamine by Fe(III) or MnO_2_. These reactions can occur over a broad range of soil characteristics but are disregarded in much current research on nitrogen cycling. Indeed, it is difficult to discriminate between biological and abiotic sources because both processes can proceed at the same time in the same soil layer [34].

### 3.2. Biological NO Synthesis via NO Synthases

Whatever the routes over geological timescales for NO evolution on Earth, an equally important consideration for “NO evolution” is the contemporary biosynthesis of NO in microbes and higher organisms. Due to its short half-life, dependent on the biological milieu (2 ms–2 s in vivo) [35], NO must be produced spatially close to its site of action. The main production of NO in cells is catalysed by NO synthases (NOS) at appropriate magnitudes and tempi. However, as discussed in Section 2, the earliest source of biologically useful NO may have been produced via non-biological processes, i.e., strictly chemical.

In mammals, besides alternative pathways to generate NO by the reduction of NO_2_^−^ and NO_3_^−^ at a low concentration of O_2_ [36,37,38,39,40], endogenous NO is mainly produced by mammalian NOS (mNOS) [41]. However, NOS-like enzymes have also been found in all three Domains of life: Archaea, Bacteria and Eukaryotes, pointing to an essential and conserved role of NO through evolution [42,43,44,45,46].

There are three isoforms of mNOS enzymes: neuronal NOS1 (nNOS) and endothelial NOS3 (eNOS)—known as the constitutive forms assigned to produce a steady-state NO level—and a third one, the inducible form NOS2 (iNOS), regulated in response to an inflammatory signal [47], for example, in macrophages. Initially classified according to their localisation, mNOS isoforms have been found in many other tissues and cells [47,48,49]. All three mNOS isoforms catalyse NO production starting from L-arginine and O_2_ as substrates and producing L-citrulline and NO. Each NOS, a homodimer, is made of a C-terminal reductase domain and an N-terminal oxygenase domain that are linked via a calmodulin-binding helix. The reaction requires as cofactors nicotinamide adenine dinucleotide phosphate reduced (NADPH), flavin mononucleotide (FMN), flavin adenine dinucleotide (FAD) and (6R-)5,6,7,8-tetrahydrobiopterin (BH_4_) [47]. The reaction mechanism has been the subject of intensive study, and its details are beyond the scope of this account. In brief, one cycle of NOS activity comprises two independent monooxygenase reactions. The first generates N-OH-L-Arg, which is the substrate for the second reaction. The role of the biopterin cofactor is now becoming clearer, implicating one-electron redox cycling as well as multiple allosteric effects on enzyme activity [50]. 

Among microorganisms, NOS enzymes have been found in many bacterial strains, including *Nocardia* [51], *Lactobacillus fermentum* [52], *Staphylococcus aureus* [53], *Bacillus subtilis* [54,55,56], *B. cereus* [57], *B. anthracis* [46], *Deinococcus radiodurans* [58], *Sorangium cellulosum* [59], *Streptomyces turgidiscabies* [60] and *Geobacillus stearothermophilus* [61], and in the haloalkaliphilic archaeon *Natronomonas pharaonis* [62] and the cyanobacterium *Synechococcus* PCC 7335 [63].

As in the NOS of higher organisms, bacterial NOS (bNOS) enzymes catalyse the oxidation of arginine to citrulline and NO in the presence of BH_4_ or tetrahydrofolate (H_4_F) [64] as cofactors. The oxygenase domain of bNOS shares 45% amino acid sequence identity with the oxygenase domain of mNOS [46]. However, bNOS is smaller than its mammalian counterparts, lacking the N-terminal domain, the CaM-binding site and the reductase domain, the last being essential for electron transfer during NO biosynthesis. However, bNOS is able to generate NO in vivo using available cellular reductases [46]. One exception is the bNOS from the Gram-negative *S. cellulosum*, which contains both a reductase and an oxygenase domain in the same polypeptide chain, with a 2Fe-2S ferredoxin subdomain involved in electron transfer from NADPH to haem [59].

bNOS enzymes seem to have different functions from those of mNOSs, including cytoprotection against oxidative stress [65], nitration of different metabolites [66], recovery from exposure to UV light [67] and modulation of aerobic respiratory metabolism [68]. bNOS may also be considered as a therapeutic target, and bNOS inhibitors may be used to potentiate the activity of some traditional antibiotics [64], although the interplay between NO and antibiotic efficacy is complex, and examples of both inhibition and potentiation of antibiotic lethality exist (see Section 7 below).

In an evolutionary context, it is reasonable to think that, in higher eukaryotes, mNOS evolved from simpler single-domain bacterial homologs, acquiring a more specialized functioning with a complex multidomain protein regulated by a specific reductase and CaM-binding domains. Furthermore, there is additional evidence that NOS evolution has been influenced by horizontal gene transfer [69,70,71,72].

## 4. Molecular Architecture of NO-Binding Sites in Proteins

### 4.1. Haem-Binding Proteins

**Figure 2:**
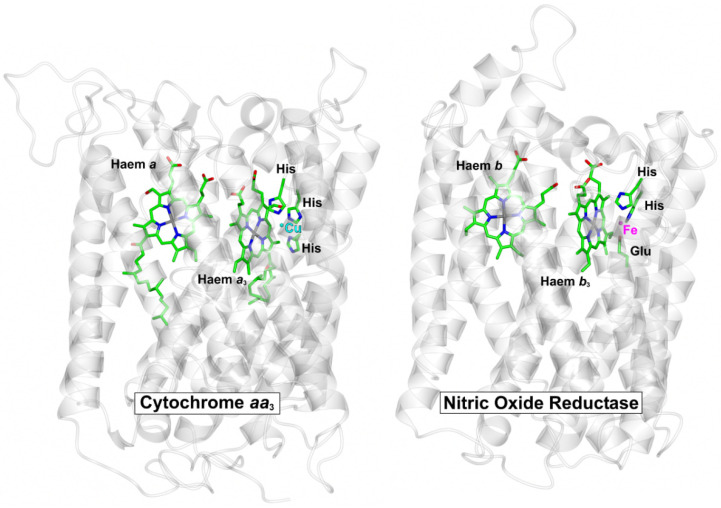
Core structures of O_2_-binding cytochrome *aa*_3_ and NO-binding cNOR complex. The protein backbones for the core catalytic subunits for cytochrome *aa*_3_ from *P. denitrificans* (pdbID = 3HB3, [83]) and the cNOR NO reductase from *P. aeruginosa* (pdbID = 3O0R, [75]) were superposed (RMSD = 2.88 Å). The central haem cofactors and the catalytic copper (Cu) and iron (Fe) centres are shown along with the sidechains that coordinate these metals. Peripheral subunits, cofactors and solutes have been omitted for clarity.

Respiratory complexes are well known to be targets for NO binding. The best characterised examples are animal and microbial globins and the terminal respiratory oxidase complexes; the latter can be classified into subgroups in a number of different ways, but arguably, the best characterised class of terminal oxidase in the context of NO binding is the haem-copper O_2_ reductases (HCOs). The HCO superfamily is classified into several sub-groups [73]; all possess a low-spin haem and a binuclear centre that comprises a high-spin haem and a copper ion ligated by three histidine residues. While the copper site can bind to NO (see below), NO will bind with high affinity to the axially liganded ferrous high-spin haem (*a*-type or *o*-type) in competition with O_2_, as reported for cytochrome *bo*’ of *Escherichia coli* [74]. Also part of the HCO superfamily are the NO reductase (NOR) complexes, which catalyse the reduction of NO to nitrous oxide and water and possess a binuclear centre that is comprised of a high-spin haem and an iron ion (Fe_B_) that is coordinated by histidine and glutamate sidechains [75]. The core structure is very similar to O_2_-binding HCOs, as illustrated for cNOR of *Pseudomonas aeruginosa* and cytochrome *aa*_3_ of *Paracoccus denitirifcans* (Figure 2), which clearly reflects the evolutionary relationship between these membrane complexes.

Globins such as haemoglobin and myoglobin are also well-known to ligate NO via the haem iron O_2_-binding site and can both scavenge NO by oxidizing it to nitrate and generate NO in vivo via the reduction of nitrite ions by the ferrous haem cofactor [26], in addition to their primary roles as O_2_ carriers. However, perhaps more relevant in terms of NO binding are the specialist NO-detoxifying globins of microorganisms [5], with Hmp representing the archetypal example [76]. The *b*-type haem cofactor of Hmp upon first inspection is a five-coordinate species similar to myoglobin, although a hydrogen-bonding network in the proximal pocket and charged residues in the distal cleft [77,78] confer properties more suitable to NO detoxification and O_2_ chemistry instead of O_2_ transport.

Other roles for NO-binding haem moieties include sensing roles within H-NOX (haem-nitric oxide O_2_ binding) domains [79,80] and as cofactors for bacterial transcription factors, such as the dissimilative NO_3_^−^ respiration regulator DNR [81] or the nitrite reductase and nitric oxide reductase regulator NNR [82]. As discussed below (Section 7), feedback loops often feature in gene regulation achieved by these regulators. H-NOX domains are often linked to other functional domains, including histidine kinases (HKs), diguanylate cyclases (DGCs) or methyl-accepting chemotaxis proteins (MCPs), and the haem-binding transcription factors obviously contain DNA-binding domains. These NO sensors derive their novel functionality via modulation of these linked domains but bind to NO via the haem iron in much the same way as other haemproteins do.

### 4.2. Non-Haem Iron Centres

Fe-S clusters also bind NO, as reported for the 2Fe-2S and 4Fe-4S clusters of the bacterial transcription factors NsrR and FNR [84,85]. Exposure of these transcription factors to NO results in the formation of dinitrosyl iron species and alleviation of transcriptional repression. Similarly, a mononuclear iron site in the bacterial transcriptional activator NorR is able to bind NO to form a mononitrosyl complex that activates the transcription of a NO-detoxifying flavorubredoxin complex [86,87]. Furthermore, the Fe_B_ site of NOR mentioned above represents another example of a non-haem iron centre that binds NO (Section 4).

### 4.3. Copper Centres

NO has been used as a probe to investigate a wide variety of copper-binding proteins that are not generally thought to encounter NO [88], including bacterial dissimilatory nitrite reductases (i.e., Nir complexes) with mononuclear copper sites; the O_2_ transport protein haemocyanin with two histidine-coordinated copper ions that can bind O_2_; and the O_2_-consuming phenoloxidases, such as tyrosinase, that also possess two histidine-coordinated copper ions.

However, perhaps more intriguing is the ability of NO to participate in redox chemistry with copper sites in a physiologically relevant way. A well-characterised example is that of NO-mediated inhibition at the Cu_B_ site of mitochondrial cytochrome *c* oxidase, where the Cu^2+^ form is thought to be reduced to Cu^+^, with concomitant production of a nitrosonium (NO^+^) intermediate and subsequent evolution of nitrous acid [88].

### 4.4. Thiols

The cellular targets of NO and its derivatives are diverse, as exemplified by the modification of thiol groups (Section 1) that are described by the unique and extensive *S*-nitroso proteomes of different cell types. One well-studied example is complex I (NADH dehydrogenase) of the respiratory chain, where *S*-nitrosylation of cysteine sidechains is thought to inhibit this complex [89]. In addition, low-molecular-weight thiols, such as glutathione, cysteine and homocysteine, are also targets for NO [4].

## 5. Evolution of NO-Binding Sites

Evolutionary histories of enzymes involved in chemiosmotic energy conversion indicate that a strongly oxidizing substrate was available to the last universal common ancestor (LUCA) before the divergence of Bacteria and Archaea. Based on recent phylogenetic, enzymatic and geochemical results [90], Ducluzeau and colleagues propose that in the earliest Archaean, NO and its derivatives NO_3_^−^ and NO_2_^−^ served as strongly oxidizing substrates driving the evolution of a bioenergetic pathway related to modern dissimilatory denitrification. As discussed below, aerobic respiration perhaps arose later via adaptation of the enzyme NO reductase to its new substrate, O_2_ gas.

The evolution of NO-binding sites in proteins is inextricably linked to a variety of external factors due to (i) the promiscuity of NO-binding sites (e.g., to also bind O_2_), (ii) the requirement for a range of cellular machinery (e.g., haem availability), and (iii) the availability of suitable metals in the correct redox state. Regarding ligand promiscuity, early homology analyses suggested that NORs were the evolutionary ancestor to the closely related O_2_-binding HCOs [91], and it is certainly intriguing that NORs have been shown to reduce O_2_ [92] and HCOs have been identified with NO-reducing capabilities [93,94]. However, without access to genomic data for LUCA, it is not possible to confidently assign a precise point of evolutionary divergence between these enzyme classes [73]. It is worth noting that molecular phylogenetic studies support the hypothesis that HCO complexes were present in the last common ancestor to archaea and bacteria, whereas oxygenic photosynthesis “appeared” later in bacteria [95,96]. Furthermore, it is thought that this last common ancestor was equipped with a range of respiratory chains that could support growth in a variety of environmental conditions, including the NOR enzyme of the denitrification pathway [97]. A comprehensive review of the structure/function of the HCO/NOR enzyme classes, along with palaeogeochemical and thermodynamic information, further supports this model for an early evolutionary divergence of O_2_-reductases and NORs prior to the Great Oxidation Event [98].

There is plenty of evidence to support the emergence of NOR complexes before oxygenic photosynthesis, so it is of interest to consider the availability of NO in an O_2_-depleted early atmosphere for use as a respiratory electron acceptor (as is the case for contemporary NO reductases). Prior to the emergence of oxygenic photosynthesis, it is thought that the oceans and atmosphere of Earth were reducing, suggesting that multivalent metals would exist mainly in their lower redox states, making them poor candidates as dissimilative electron acceptors. Hence, the availability of alternative electron acceptors is likely to have been less diverse than today, making NO a more attractive electron acceptor in the absence of more modern competition (e.g., O_2_, ferric iron) [99,100]. These primordial reducing conditions may also have presented a hurdle for the incorporation of copper ions (but not ferrous iron) into NO-binding sites due to the propensity of reduced cuprous Cu^+^ ions to disproportionate, although it has been suggested that coordination of Cu^+^ by appropriate ligands (e.g., thiolates) can stabilize this ionic state [101]. Furthermore, the presence of NO-binding copper centres in obligate anaerobes (e.g., HCOs) demonstrates that copper is available for incorporation in anoxic environments. Hence, these observations support the idea that, prior to the Great Oxidation Event, NO was present alongside the suitable metallic precursors to generate simple NO-binding centres. It has been hypothesized that, following the increase in atmospheric O_2_, copper O_2_ proteins (COPs) with simple copper centres (e.g., the phenoloxidase tyrosinase) evolved to remove atmospheric O_2_ [102,103], and it is intriguing, yet perhaps unsurprising, that these protein family members can also bind NO [88].

More complex NO ligands, such as haems, are synthesised by contemporary organisms via highly conserved haem biosynthetic pathways or can be acquired directly from host organisms, although for the latter route, the haem is often used only as an iron source. However, evidence exists for the abiotic synthesis of metalated porphyrins under conditions that simulate primordial volcanic rock pools [104], suggesting that it may have been possible for NO-binding haems to have evolved prior to the widespread oxygenation of the atmosphere. However, to suggest that proteins may have evolved NO-binding sites independently of O_2_ remains highly speculative. More recently, geologically speaking, the equilibrium of NO within the nitrogen cycle is maintained by a diverse array of haem-dependent processes (e.g., NO production via bacterial nitrite reductase), so the utilisation of atmospheric NO by current biological processes is now dependent upon haem biosynthesis rather than a potential abiotic haem source in the primordial milieu.

## 6. Biological Roles for NO

The biology of NO should be considered in parallel with other small molecules (“gasotransmitters”) that may also be gaseous, specifically CO and H_2_S. All three may be endogenously generated via regulated processes, they function at exceptionally low concentrations, and each can be toxic. The evolution of NO that preceded life presented numerous opportunities for beneficial employment of this gas (as well as potentially toxic consequences, as discussed in Section 1). These roles are dependent on those features common to all “gasotransmitters”, as follows. A gasotransmitter is defined as a small, generally reactive, gaseous molecule that, in solution, is generated endogenously in an organism and exerts important signalling roles. It is noteworthy that these molecules are also toxic and antimicrobial. Recent advances show that, as in higher organisms, bacteria can synthesise each of these gases, but the molecules also have important signalling or messenger roles in addition to their toxic effects. However, strict application of the criteria proposed for a gasotransmitter leads some authors to conclude that the term “small molecule signalling agent”, as proposed by Fukuto and others, is preferable terminology [105].

The NO signalling pathway is spread throughout the entire phylogenetic scale [42,48]. In contrast to CO and H_2_S, the information related to NO signalling is overwhelming in the literature showing an unprecedented burst of activity after the discovery of NO as an endogenous vasoactive molecule. Here, we mention only briefly the well-understood role for NO in cell signalling with emphasis on marine invertebrates and fish that are continuously exposed to changing environmental conditions and where NO serves in a wide variety of physiological functions.

In the classical NO signalling pathway, NO binds the haem of sGC to promote the conversion of guanosine-5′-triphosphate (GTP) to cyclic guanosine monophosphate (GMP) for the downstream activation of protein kinase G; cGMP signalling is fundamental in cardiovascular physiology [106] and in neurotransmission. When eNOS is activated, newly synthesized NO diffuses into adjacent smooth muscle cells to promote the formation of cGMP, which induces muscle relaxation by mechanisms that activate protein kinase activity with consequent phosphorylation of myosin light chains [107].

The origins and actions of NO, CO and H_2_S in mammalian physiology have attracted most of the attention for the variety of homeostatic functions performed by these gases in many cells and tissues in health and disease. The nature of NO as a signalling molecule was revealed through extensive scientific research on mammalian models, which implicated NO in a number of major cellular functions, including cell proliferation, differentiation, apoptosis, macrophage activity and neurotransmission [47,108,109]. However, studies of non-mammalian models (particularly fish and marine invertebrates) provide excellent examples to understand its evolutionary role in signalling processes [17]. In marine invertebrates, many of the effects of NO are mediated, as in mammals, by the classical NO signalling pathway and are mostly related to defence [110,111], environmental stress [112,113,114], control of swimming [115], symbiosis [116,117] and larval settlement and metamorphosis [118,119]. Marine invertebrates are endowed with distinct life cycles, and some undergo a metamorphic transition from pelagic larvae to benthic juvenile. Larval metamorphosis is a dynamic, biological environmentally dependent process in which NO signalling plays fundamental roles by regulating metamorphosis timing [120,121].

In many fish, NO (or NO donors in vitro) may act as vasodilators by the classical NO signalling pathway acting via sGC. However, unlike in mammals, NO does not appear to have an endothelial origin in fish, as the endothelial NOS appears lost in all teleost genomes annotated to date [108,122]. Therefore, the endothelial NO signalling mediated by the isoform NOS3 is not ubiquitous in non-mammalian vertebrates, being found only in reptiles, birds and mammals. Some data obtained from perfused tissue preparations contrast with the evidence that, in teleosts, the endothelial signalling is absent [123]. Neural NOS is prevalent in perivascular nerves and is the most likely source of NO for cardiovascular control in fish [108,122]. The recent completion of the annotated genome of the non-teleost actinopterygian *Lepisosteus oculatus* [124] reveals the presence of three different *NOS* genes encoding NOS1, NOS2 and NOS3 proteins, respectively [122]. The *L. oculatus* lineage diverged from teleosts before teleost genome duplication (TGD). In summary, most of the evidence clearly indicates that, in lower vertebrates, NO does not play a role as a vasodilator, and in many species, it does not affect vascular tone. In cyclostomes, NO has been found to be a vasoconstrictor and a vasodilator. In chondrichthyan fishes, the vasodilation mediated by NO was absent [122].

An NOS enzyme was also found in the haemocytes of the American horseshoe crab (*Limulus polyphemus*) [125]. The NO produced by NOS was able to downregulate the aggregation of haemocytes as described in mammalian platelets. These data suggest that formation of NO from L-arginine ranks among the oldest regulatory systems since the horseshoe crab is an ancient species subjected to very little morphological change over 500 million years of evolution. The origin of NOS in metazoans and the evolutionary events responsible for their functional and structural specialization are still open questions [47]. Recurrent gene and genome duplication events might have led to the independent acquisition of novel functions in many lineages [47]. These duplications might have allowed acquisition of different specializations in different animal lineages with some invertebrate NOS proteins able to play joint roles.

However, NO has many biological functions that are independent of sGC and cGMP. These include the direct regulation of ion channels, the modulation of mitochondrial enzymes and post-translational modifications of proteins by *S*-nitrosylation, *S*-glutathionylation, tyrosine nitration, and *S*-guanylation [126]. The biological actions of NO in non-mammalian vertebrates are probably also mediated by similar mechanisms. As in mammals, NO from bacterial bNOS activity is linked to two post-translational modifications, *S*-nitrosylation [127,128] and nitration [60,129].

The major post-translational modification is the *S*-nitrosylation of proteins by covalent modification of thiols. Aberrant levels of *S*-nitrosylation are detrimental to the cell and result in altered redox state and pathological conditions [130]. The redox status of the cell influences NO signalling: in the presence of low levels of reactive-O_2_ species (ROS), S-nitrosylation not only scavenges NO to prevent reaction with ROS, but also protects cysteine thiols from oxidation [131].

## 7. Roles for NO Evolution during Infection and Prospects for Drug Development

NO is able to modulate many physiological responses in all organisms, including gene regulation, cytostasis, apoptosis, platelet function, vascular smooth muscle cell relaxation and proliferation, neurotransmission, memory, and immune stimulation. Due to its lipophilic chemistry, NO traverses cell and organelle membranes to modulate downstream signalling functions. The redox chemistry central to NO-based signalling plays essential roles in biological systems by supporting life under physiological and physio-pathological conditions. Therefore, many diseases are characterised or associated with perturbations in NO production/signalling pathways. During microbial infection, macrophages and neutrophils upregulate an iNOS that produces toxic levels of NO to combat infection. The iNOS enzyme is upregulated at the transcriptional level in response to bacterial lipopolysaccharide (LPS), which modulates its effects via activation of Toll-Like Receptor signalling. NO is then generated by iNOS using L-arginine as a substrate, which diffuses into and across the bacterial membrane and exerts its toxic effects as a defence against infection [2]. The ability of NO to inhibit microbial respiration is detailed above. In addition to respiratory inhibition, NO also reacts directly with Fe-S clusters. It is notable that the reaction of NO with Fe-S clusters and haems can lead to negative loops of feedback regulation, for example, in the control of flavohaemoglobin gene expression [132]. The derivatives that result from spontaneous reaction with O_2_ (NO_2_; N_2_O_3_; N_2_O_4_) can modify tyrosine sidechains, DNA bases and thiol groups [133]. The reaction of NO with O_2_^−^ to form ONOO^−^ (Section 1) can result in the nitration of protein tyrosine residues [134]. Tyrosine nitration is a form of protein post-translational modification that is predominantly non-enzymatic and may be observed under conditions of nitrosative stress and in certain disease states. Exposure of bacteria to NO can elicit wide-ranging effects via modulation of redox poise via nitrosylation of low-molecular-weight thiols (e.g., glutathione) and through modification of a broad array of thiol groups on the cysteine sidechains of various proteins (i.e., the *S*-nitroso proteome) [135,136]. Note that the reversal of protein nitration by an activity named denitrase has also been described [137]. Thus, tyrosine nitration of specific proteins is reversible and may contribute to signal transduction. A small protein motif (14–18 amino acids) responsive to tyrosine nitration has been developed by Urmey et al. [138].

The discovery of the important signalling role for NO in the cardiovascular and nervous systems in the 1980s began to explain why organic NO_3_^−^ (e.g., nitroglycerin) and sodium nitroprusside had long been effective treatments for angina and hypertension. Subsequently, research into NO donors as therapeutics intensified, and several thousand publications accumulated over the next few decades. The flurry of research activity that followed the 1998 Nobel prize yielded several classes of NO-releasing drugs, although the diversity of NO-releasing drugs used in a clinical setting remained limited to organic NO_3_^−^ and sodium nitroprusside even a decade after the key discoveries for the Nobel prize were made [139]. That said, a host of other NO-releasing drugs have been developed with different chemistries and half-lives [140], including diazeniumdiolates (NONOates), SNOs, hybrid compounds that link NO releasers to non-steroidal anti-inflammatory drugs or aspirin, insoluble microporous “zeolites” that release NO when hydrated, and more recently, the development of a variety of light-induced NO releasers [141,142,143]. NONOates are comprised of a diolate group [N(O−)N=O] bound to a nucleophile adduct and will decompose spontaneously in solution at physiological pH in a manner that is independent of thiols or the biological tissue, making the rate of NO release predictable. For this reason, they are attractive drugs to treat cardiovascular disease and have been used to prevent thrombosis and neointimal formation that can result from vascular injury [144]. Hybrid NO-donor drugs are part of a diverse family where approved drugs are derivatised to contain NO-releasing moieties. A number of such hybrids have been generated using aspirin as the parent compound, the aim being that NO would counteract adverse gastrointestinal symptoms that can result from prolonged treatment of inflammatory disorders with aspirin alone. Indeed, NO–aspirin compounds have been shown to provide anti-inflammatory benefits without the formation of gastric ulcers that are caused by comparable exposure to aspirin alone [145]. Applications for light-activated NO releasers are still in their infancy, although promising work has been reported on vascular relaxation in rats [142,146,147].

With regard to research on NO as an antimicrobial agent, there are several hundred articles dedicated to the well-known toxic effects of NO, well beyond the scope of this article. However, perhaps a more interesting future trend is the combinatorial treatment of NO with conventional antibiotics. The evolution of NO from chemical donors has recently been shown to potentiate the effects of antibiotics, including the outer membrane disruptor colistin, the aminoglycoside tobramycin, meropenem and the beta-lactam aztreonam in *P. aeruginosa* [148]. In addition, NO gas has also been reported to enhance the lethality of clofazimine and amikacin towards *Mycobacterium abscessus* [149]. However, NO has previously been shown to potently diminish the lethality of a number of aminoglycosides (including tobramycin) towards *Salmonella* [150], and NO donors have been shown to protect *E. coli* against gentamicin lethality under both aerobic and anaerobic conditions [151]. Furthermore, endogenously produced NO (i.e., via a bNOS enzyme) has been shown to protect Gram-positive bacteria against a broad array of antibiotic classes [152]. Clearly, the interplay between antimicrobial action and NO is complex, and the molecular basis of these effects is not fully understood, although it is probable that this relationship will be influenced by the bactericidal mode of action of the antibiotic and also the physiological state of the cell. Indeed, compounds that decelerate respiratory metabolism have previously been shown to elicit a profound decrease in lethality for a range of antibiotics against a number of bacterial pathogens [153], which could explain the observed antagonistic relationships between NO exposure and the efficacy of certain antibiotics (e.g., gentamicin). The conflicting reports of NO as both a potentiator and inhibitor of antibiotic action could be explained by different loads of NO exposure affecting the metabolic activity of different bacterial species to varying degrees, and different antibiotics requiring disparate metabolic processes to execute their lethal mode of action.

## 8. Conclusions

The inherent chemical reactivity of NO poses significant problems in any attempt to understand the development of its roles and toxicity in biology over evolutionary timescales. Whatever its mode of appearance in the early atmosphere may have been (from volcanic or other sources, as discussed here), we cannot know with certainty how its reactions with biotic and abiotic targets contributed to it becoming such a critical and ubiquitous signalling agent and toxin. Of special note is the fact that it probably evolved in the Earth’s atmosphere before oxygen, suggesting that NO-reducing, rather than oxygen-reducing, enzymes may have been early contributors to terminal electron transport processes and energetics. In other words, aerobic respiration probably arose later than the evolution of NO in the atmosphere via adaptation of the enzyme NO reductase to its new substrate, O_2_ gas. The reactivity of NO (a radical species) challenged development of all lifeforms; we and others speculate that the earliest biological roles of contemporary haemoproteins (notably haemoglobins) may have been NO detoxification. In this short review, we survey the chemical congeners that arise from the diverse reactions of NO in biological milieu, outline the many types of biological processes with which NO may interact, and discuss the roles of NO in numerous signalling mechanisms in biology. Given the voluminous literature on NO signalling, we only briefly review some of the current biological roles for NO in microbes, invertebrates and higher organisms. Finally, we speculate on how understanding NO toxicity may contribute to the fight against antibiotic-resistant bacteria.

## Data Availability

Not applicable.
